# Enigma Prevents Cbl-c-Mediated Ubiquitination and Degradation of RETMEN2A

**DOI:** 10.1371/journal.pone.0087116

**Published:** 2014-01-23

**Authors:** Stephen C. Kales, Marion M. Nau, Anand S. Merchant, Stanley Lipkowitz

**Affiliations:** 1 Women’s Malignancies Branch, Center for Cancer Research, National Cancer Institute, National Institutes of Health, Bethesda, Maryland, United States of America; 2 Center for Cancer Research Bioinformatics Core, Advanced Biomedical Computing Center, SAIC-Frederick, Frederick, Maryland, United States of America; Loyola University Chicago, Stritch School of Medicine, United States of America

## Abstract

The Cbl proteins (Cbl, Cbl-b, and Cbl-c) are a highly conserved family of RING finger ubiquitin ligases (E3s) that function as negative regulators of tyrosine kinases in a wide variety of signal transduction pathways. In this study, we identify a new Cbl-c interacting protein, Enigma (PDLIM7). This interaction is specific to Cbl-c as Enigma fails to bind either of its closely related homologues, Cbl and Cbl-b. The binding between Enigma and Cbl-c is mediated through the LIM domains of Enigma as removal of all three LIM domains abrogates this interaction, while only LIM1 is sufficient for binding. Here we show that Cbl-c binds wild-type and MEN2A isoforms of the receptor tyrosine kinase, RET, and that Cbl-c enhances ubiquitination and degradation of activated RET. Enigma blocks Cbl-c-mediated RETMEN2A ubiquitination and degradation. Cbl-c decreased downstream ERK activation by RETMEN2A and co-expression of Enigma blocked the Cbl-c-mediated decrease in ERK activation. Enigma showed no detectable effect on Cbl-c-mediated ubiquitination of activated EGFR suggesting that this effect is specific to RET. Through mapping studies, we show that Cbl-c and Enigma bind RETMEN2A at different residues. However, binding of Enigma to RETMENA prevents Cbl-c recruitment to RETMEN2A. Consistent with these biochemical data, exploratory analyses of breast cancer patients with high expression of RET suggest that high expression of Cbl-c correlates with a good outcome, and high expression of Enigma correlates with a poor outcome. Together, these data demonstrate that Cbl-c can ubiquitinate and downregulate RETMEN2A and implicate Enigma as a positive regulator of RETMEN2A through blocking of Cbl-mediated ubiquitination and degradation.

## Introduction

Receptor Tyrosine Kinase (RTK) signaling is essential for normal biological processes and disruption of this regulation can lead to tumor initiation and progression. Cbl proteins are a family of RING finger ubiquitin ligases (E3) that negatively regulate a variety of RTKs (including EGFR, MET, and RET), tyrosine kinase dependent receptors such as the T-cell receptor, and non-receptor tyrosine kinases such as Src. There are three mammalian Cbl proteins: Cbl (a.k.a., c-Cbl, Cbl2, and RNF55), Cbl-b (a.k.a., RNF56), and Cbl-c (a.k.a., Cbl-3, Cbl-SL, and RNF57) [1], [2]. Cbl proteins have a highly conserved N-terminus consisting of a tyrosine kinase binding (TKB) domain that binds to specific phosphorylated tyrosines on substrates, a catalytic RING Finger (RF) domain, and an alpha helical linker region separating the TKB and RF domains [1], [2]. The C-termini of the Cbl proteins are the least conserved; however, each is comprised of a proline rich (PR) region which mediates interactions with SH3-domain containing proteins. The C-termini of Cbl and Cbl-b share tyrosines that, upon phosphorylation, serve as sites of SH2 protein interaction [3] as well as a ubiquitin associated (UBA) domain which has been shown to mediate homodimerization and ubiquitin binding [4], [5].

While Cbl and Cbl-b have been well studied and characterized, less is known about Cbl-c. Unlike the ubiquitous distribution of Cbl and Cbl-b, Cbl-c is expressed exclusively in epithelial cells [6]–[9]. Like Cbl and Cbl-b, the N-terminus of Cbl-c is composed of the highly conserved TKB, RF, and linker region, but Cbl-c is the most divergent of the three proteins from the Cbl sequence [2], [7]. The C-terminus of Cbl-c diverges from the other two Cbl proteins by its shorter PR domain, absence of C-terminal tyrosines, and lack of a UBA domain. Cbl-c, like Cbl and Cbl-b, is a functional E3 that can ubiquitinate and downregulate EGFR and v-Src in cells [7], [10], [11]. Mice null for Cbl or Cbl-b have clear immunological and hematological defects that help to define their physiological roles; however, mice null for Cbl-c are viable and fertile with no apparent abnormalities [6], [12]–[15]. Thus, the physiological role of Cbl-c is not clear.

To elucidate the function of Cbl-c, we sought to determine Cbl-c interacting proteins by utilizing a yeast two-hybrid screen and subsequently identified Enigma (a.k.a., PDLIM7) as a Cbl-c binding partner. Enigma is a member of the LIM family of proteins and is comprised of an N-terminal PDZ domain, which has been shown to mediate actin filament binding [16], [17], and three C-terminal LIM domains, which mediate protein-protein interactions [18]. LIM1 is the least conserved of the three LIM domains and thus was excluded from the original characterization of Enigma and has no known interacting partner [19]. LIM2 and LIM3 (originally LIM1 and 2) have been shown to bind RET [20] and InsR [19] respectively. RET (*REarranged during Transfection*) is an RTK which binds members of the glial cell line-derived neurotrophic factor (GDNF) family of extracellular signaling molecules [21]. RET loss of function mutations are associated with the development of Hirschsprung’s disease [22] while gain of function mutations or translocations are associated with the development of a variety of human cancers, including medullary thyroid carcinoma, multiple endocrine neoplasias (MEN) type 2A and 2B, pheochromocytoma, and parathyroid hyperplasia [23], [24]. Previous reports demonstrate Enigma as a required component for mitogenic signaling of RET/PTC [16], a rearranged oncogenic isoform of RET found in papillary thyroid carcinomas. In general, Enigma is considered a scaffold protein which, through an actin binding PDZ domain and three protein-binding LIM domains, serves as an adaptor to stabilize membrane associated signaling complexes [17]. Here, we show that Enigma is a binding partner of Cbl-c and that through its interactions with both Cbl-c and RET blocks RET ubiquitination and degradation thereby serving as a positive regulator of the oncogenic RETMEN2A isoforms.

## Materials and Methods

### Reagents

Dulbecco’s modified Eagle’s medium (DMEM), fetal bovine serum (FBS), penicillin, and streptomycin sulfate were obtained from Invitrogen (Carlsbad, CA). Dulbecco’s phosphate buffered saline (D-PBS) was purchased from Mediatech Inc. (Herndon, VA). Tissue culture plastic ware and other laboratory consumables were purchased from commercial sources. Recombinant human EGF (#354052) was obtained from BD Biosciences (Bedford, MA). MG-132 (#474790) was purchased from Millipore (Billerica, MA), and cycloheximide (#C7698) was purchased from Sigma Aldrich (St. Louis, MO). Recombinant human RET ligand (GDNF; #714-GR-100) and exogenous co-receptor (GFRα1; #212-GD-010/CF) were purchased from R&D Systems (Minneapolis, MN).

### Expression Constructs

The expression plasmids for full length HA epitope tagged Cbl, Cbl-b, Cbl-c, and the control vector (pCEFL) have been described previously [7], [25]. The full-length human Enigma cDNA construct was purchased from OpenBiosystems (MHS4771-202828331; Huntsville, AL). FLAG-Enigma was constructed using PCR and sub-cloned into pcDNA3.1. Point mutation and deletion constructs described above were created using the QuikChange II Site-directed Mutagenesis Kit according to manufacturer’s instructions (Stratagene, La Jolla, CA). Plasmids encoding human RET9, RET51, their corresponding MEN2A (C634R) mutants and the GFRα1 co-receptor were kindly provided by Carlos Ibanez [26]. RET9MEN2A point mutation and deletion constructs were made using site-directed mutagenesis. All constructs were confirmed by DNA sequencing.

### Yeast Two-hybrid Screen

Yeast two-hybrid screening was carried out by Myriad Genetics (Salt Lake City, UT), using a partial human Cbl-c cDNA (representing amino acids 360–474) as the bait in a mating-based screening method. The Cbl-c cDNA was cloned into pGBT.superB creating an open reading frame for Cbl-c fused to the GAL4 DNA-binding domain. The bait plasmid was introduced into Myriad’s ProNet yeast strain PNY200 (MATα *ura*3-52 *ade*2-101 *trp*1-901 *his*3-Δ200 *leu*2-3112 *gal*4Δ *gal*80Δ). Bait yeast cells were mated with Myriad’s ProNet MATa yeast cells, BK100 (MATα *ura*3-52 *trp*1-901 *his*3-Δ200 *leu*2-3112 *gal*4Δ *gal*80Δ GAL2-ADE2 LYS2::GAL1-HIS3 *met*2::GAL7-*lac*Z) containing three independent cDNA libraries from human breast/prostate cancer, spleen and brain. After mating, at least 5 million diploid yeast cells were obtained from each library and selected on His- and Ade-lacking medium. Prey plasmids were isolated from positive colonies, and the interaction was confirmed by expression of a third reporter gene (lacZ). Positive prey plasmid cDNAs were then sequenced.

### Cell Culture and Transfections

All cell lines used in this study were originally obtained from ATCC (Manassas, VA) and maintained in our laboratory through regular passage and cryopreservation. The human embryonic kidney (HEK293T) and the human pancreatic cancer (CFPac-1) cell lines were maintained in culture using DMEM (Gibco, Grand Island, NY) supplemented with 10% FBS, 100 U/ml penicillin, and 100 µg/ml streptomycin sulfate. The breast cancer cell lines MDA-MB-231 and T47D were maintained in culture using RPMI (Gibco, Grand Island, NY) supplemented with 10% FBS, 100 U/ml penicillin, and 100 µg/ml streptomycin sulfate. HEK293T cells were transfected using calcium phosphate (Profection; Promega Corp., Madison, WI). Transfections were incubated 18 h prior to media change and grown for a total of 48 h prior to harvesting. For cell treatments, all transfections were performed in replicate, then pooled, and re-plated prior to treatment. Each cell-based experiment was repeated at least 3 times.

### Immunoblotting and Immunoprecipitation

To harvest proteins, cells were washed twice in ice-cold Dulbecco’s-PBS containing 200 µM sodium orthovanadate (Fisher Chemicals, Fairlawn, NJ) and then lysed in ice-cold lysis buffer (10mM Tris-HCl pH 7.5, 150mM NaCl, 5mM EDTA, 1% Triton X-100, 10% glycerol, 100mM iodoacetamide [Sigma-Aldrich Corp., St. Louis, MO], 2mM sodium orthovanadate, and protease inhibitors [Complete tabs®, Roche Diagnostics Corp., Indianapolis, IN]). All whole cell lysates were cleared of cellular debris by centrifugation at 16,000×*g* for 15m at 4°C. Supernatant protein concentrations were determined using the Bio-Rad protein assay (Bio-Rad, Hercules, CA). For immunoblotting, lysates (2 µg protein/µl) were boiled in loading buffer (62.5mM Tris-HCl pH 6.8, 10% glycerol, 2% SDS, 1mg/ml bromphenol blue, 0.3573M β-mercaptoethanol) for 5 m. For immunoprecipitations, 300 µg of each of the whole cell lysates were incubated with either: a rabbit polyclonal anti-RET antibody (sc-13104; Santa Cruz Biotechnology, Santa Cruz, CA) or rabbit anti-EGFR (Ab-3; Millipore) with Protein A/G Plus-agarose beads (sc-2003; Santa Cruz Biotechnology, Santa Cruz, CA). For HA immunoprecipitations, 300 µg of whole cell lysates were incubated with HA-affinity matrix (11815016001; Roche Diagnostics Corp., Indianapolis, IN). For Cbl-c immunoprecipitations, whole cell lysates were incubated with agarose immobilized affinity rabbit anti-Cbl-c (Rockland Immunochemicals, Gilbertsville, PA). For GST pull-downs, 300 µg of each of the whole cell lysates were incubated with Glutathione Sepharose 4B (Amersham Biosciences, Piscataway, NJ). All immunoprecipitations were incubated overnight at 4°C with tumbling. Immune complexes were washed five times in 1mL cold lysis buffer, then resuspended in 2X loading buffer, boiled for 5m, then resolved by SDS-PAGE and transferred to nitrocellulose membranes (Protran BA85; Whatman, Sanford, MA). For immunoblot detection of proteins, the following antibodies were used: rabbit anti-RET (sc-13104; Santa Cruz), (C31B4; Cell Signaling), rabbit anti-EGFR (2232L; Cell Signaling), rat monoclonal high affinity anti-HA-peroxidase, (clone 3F10; Roche), mouse anti-GST (sc-138; Santa Cruz), mouse anti-Hsc70 (sc-7298; Santa Cruz), rabbit anti-GFP-FL (sc-8334; Santa Cruz), goat anti-Cbl-c (sc-8372; Santa Cruz), rabbit monoclonal anti-p44/42 MAPK (4370; Cell Signaling), and rabbit polyclonal anti-ERK2 (sc-154; Santa Cruz). Horseradish peroxidase linked donkey anti-rabbit IgG (NA934V; GE Healthcare, Piscataway, NJ), horseradish peroxidase linked donkey anti-mouse IgG (NA931: GE Healthcare, Piscataway, NJ), or rabbit anti-goat (sc-2768; Santa Cruz Biotechnology, Santa Cruz, CA) immunoglobulin was used with SuperSignal (Pierce Biotechnology Inc., Rockford, IL) to visualize target proteins. Each experiment was repeated at least 3 times. Densitometric analysis of immunoblot band intensities was performed using Adobe Photoshop software version 7.0 (Adobe Systems Inc., U.S.A).

### Data Mining, Retrieval and Survival Analysis

The Oncomine™ database was queried to observe expression profiles for RET, Cbl-c and Enigma in clinical breast cancer microarray datasets that also had survival data. Based on this analysis, two breast cancer microarray datasets: GSE25066 [27] and GSE20685 [28] were chosen for further analysis. Both the microarrays were processed on the Affymetrix HG-U133 chip and the normalized log-intensity expression values were retrieved from NCBI GEO database. The merged cohort for these datasets consisted of 835 samples. RET, Cbl-c and Enigma log-intensity values were available for this cohort. Based on a median cutoff of 8.0 for expression of RET across these samples, a subset of 459 patients were extracted that had RET expression above the median value. The Survival Risk Group Prediction module on BRB-ArrayTools (v 4.3) was used to analyze whether Cbl-c, Enigma and RET have a statistically significant association to the survival data for breast cancer patients within the chosen datasets. A supervised principal components model was used in this study to analyze the censored survival data. The evaluation of the predictive method was further validated using the 10-fold cross-validation. The Kaplan-Meier curves were plotted based on the low and high expressing groups predicted by the computations done above, with the statistical significance for survival being derived by calculating the log-rank statistical p-value based on 1000 permutations.

## Results

### Yeast Two-hybrid Screen Identifies Enigma as a Cbl-c Interacting Protein

**Figure 1:**
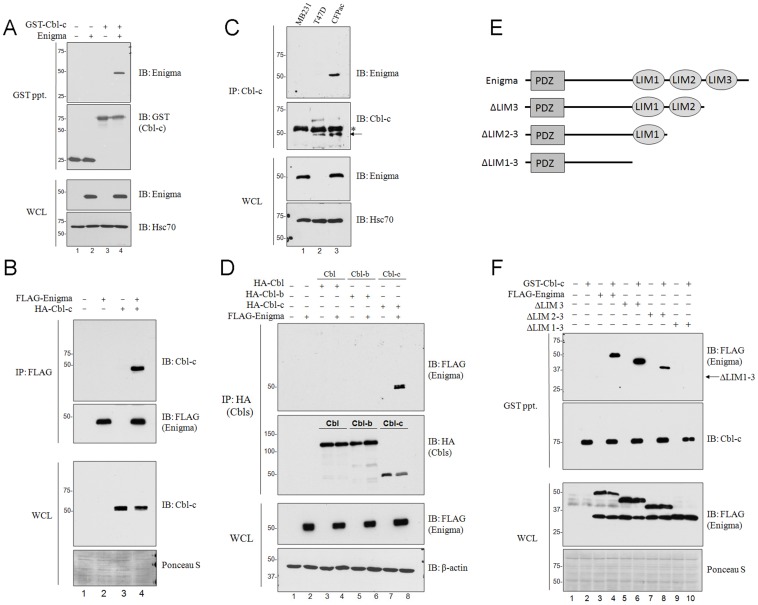
Enigma interacts with Cbl-c. (**A**) HEK293T cells were transfected with GST, or GST-Cbl-c with and without untagged human Enigma as indicated above each blot. All transfections were balanced with empty vector controls and whole cell lysates were collected at 48 h post-transfection. GST pull-downs were performed using 300 µg of whole cell lysates. GST pull-downs (GST ppt.) and whole cell lysates (WCL) were immunoblotted (IB) as indicated to the right of each blot. Molecular weight standards in kDa are indicated to the left of each panel and Hsc70 is shown as a loading control. (**B**) HEK293T cells were transfected with HA-Cbl-c with and without FLAG-Enigma as indicated above each blot. Immunoprecipitations were performed using anti-FLAG-M2 agarose with 300 µg of whole cell lysates. IPs and WCL were immunoblotted as indicated to the right. Molecular weight standards are indicated to the left of each panel and Ponceau S staining serves as a loading control. (**C**) Immunoprecipitation of endogenous Cbl-c was performed on whole cell lysates from three human cell lines: 30mg of MB-231, a triple negative breast cancer cell line; 30mg of T47D, a human ER^+^ breast cancer cell line; and 20mg of CFPac-1, a human pancreatic cancer cell line. Immunoprecipitations using rabbit anti-Cbl-c conjugated sepharose, were immunoblotted for endogenous Cbl-c (indicated by arrow) and for endogenous Enigma as indicated. Rabbit immunoglobulin is indicated with an asterisk (*) and molecular weight standards are indicated on the left of each panel. Whole cell lysates were immunoblotted for endogenous Enigma and Hsc70 which serves as a loading control. (**D**) HEK293T cells were transfected with HA-Cbl, Cbl-b, or Cbl-c with and without FLAG-Enigma. Cbl immunoprecipitations were performed on 300 µg of each of the whole cell lysates using anti-HA affinity matrix and immunoblotted for co-immunoprecipitation of Enigma, as indicated. Whole cell lysates were immunoblotted as indicated and molecular weight standards are indicated on the left of each panel. β-actin serves as a loading control. (**E**) Enigma truncation constructs used for mapping Cbl-c interaction site. (**F**) HEK293T cells were co-transfected with GST-Cbl-c along with FLAG-Enigma, ΔLIM3, ΔLIM2-3, or ΔLIM1-3. Whole cell lysates were collected and immunoblotted for Enigma constructs with anti-FLAG and for Cbl-c as indicated on the right. GST pull-downs were immunoblotted for GST-Cbl-c with anti-GST and for each of the FLAG-Enigma proteins as indicated. Arrow indicates expected position of ΔLIM1-3 protein. Molecular weight standards are indicated on the left of each panel and Ponceau S staining serves as a measure of protein loading.

We identified the LIM protein Enigma as a potential interacting protein of Cbl-c using a yeast two-hybrid screen of human cDNAs against a 114 amino acid C-terminal bait construct of human Cbl-c (encoding amino acids 360–474). To confirm this interaction, full-length cDNAs of GST-tagged human Cbl-c (GST-Cbl-c) or GST alone were co-expressed with untagged human Enigma in HEK293T cells. Subsequent GST pull-downs of GST-Cbl-c from whole cell lysates co-precipitated Enigma, but GST pull-downs of GST alone did not, thereby confirming the interaction between Cbl-c and Enigma (Figure 1A). This interaction was further validated by immunoprecipitation of FLAG-tagged Enigma which co-immunoprecipitated HA-tagged Cbl-c from whole cell lysates of HEK293T cells expressing each plasmid (Figure 1B). The interaction between endogenous proteins was established by specific co-immunoprecipitation of Enigma with immunoprecipitated Cbl-c from lysates of cells that express both proteins (CFPac-1, Figure 1C, top panel, lane 3) but not from cells that express high levels of endogenous Enigma (MB231, Figure 1C top panel, lane 1) or Cbl-c alone (T47D, Figure 1C, top panel, lane 2).

Since Cbl-c is the most divergent of the three mammalian Cbl proteins [2], [7], we next tested whether this interaction is specific to Cbl-c or is conserved among all the human Cbl proteins. To test the interaction of Enigma with each of the human Cbl proteins, we co-expressed FLAG-Enigma with HA-tagged constructs of Cbl, Cbl-b, and Cbl-c in HEK293T cells, followed by immunoprecipitation of each of the Cbl proteins with an anti-HA affinity matrix. Enigma co-precipitated with Cbl-c (Figure 1D, top panel, lane 8) but not with Cbl or Cbl-b (Figure 1D, top panel, lanes 4 and 6 respectively) indicating a selective interaction between Cbl-c and Enigma.

### The LIM Domain of Enigma is Required for Cbl-c Interaction

Enigma is comprised of three carboxyl-terminal LIM domains which serve as primary sites of protein-protein interaction [18]. LIM1 is the most divergent of the three LIM domains and was not recognized in the original characterization of Enigma. The LIM1 domain of Enigma has no known interacting partner [19], [29]. LIM2 and LIM3 have been shown to mediate binding to RET and InsR respectively [19], [20]. To determine if the interaction between Enigma and Cbl-c is mediated through LIM domain binding, we constructed a series of FLAG-Enigma constructs with C-terminal truncations to sequentially delete each of the three LIM domains, denoted as ΔLIM3, ΔLIM2-3 and ΔLIM1-3 (Figure 1E). Whole cell lysates immunoblotted for the FLAG epitope demonstrated the reduced size for each of the C-terminal truncation mutants (Figure 1F; third panel). Each of the FLAG-Enigma transfections also revealed a common band migrating to a size similar to that of the ΔLIM1-3 mutant. While this band appears to include a FLAG epitope, it did not co-precipitate with GST-Cbl-c and may represent a non-interacting product of FLAG-Enigma degradation or incomplete translation. GST pull-downs from whole cell lysates of HEK293T cells expressing GST-Cbl-c in the presence of each of the Enigma constructs revealed that Enigma co-precipitation is abrogated in the absence of all three of the C-terminal LIM domains (Figure 1F, top panel, lane 10). Enigma lacking LIM domain 3 or lacking LIM domains 2 and 3 retained Cbl-c interaction (Figure 1F, top panel, lanes 6 and 8, respectively) indicating that the region including LIM1 is sufficient for Cbl-c interaction.

### Cbl-c Binds RET Kinase

**Figure 2:**
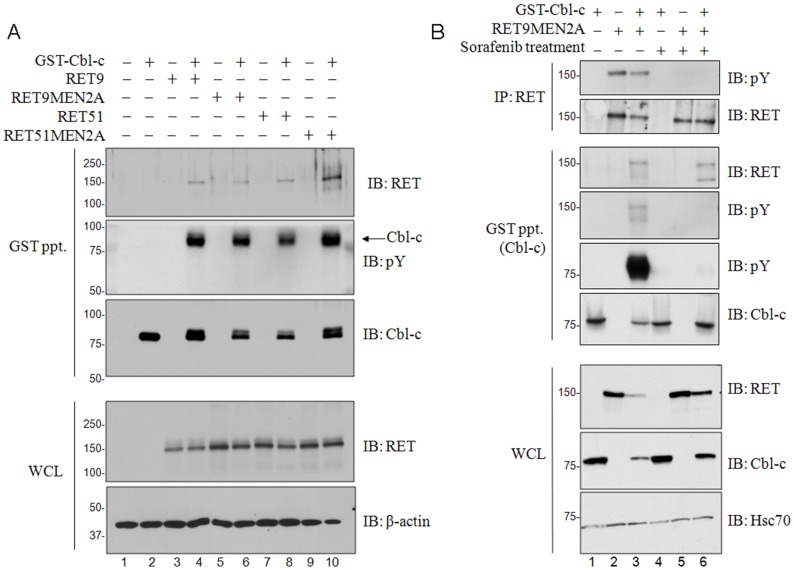
Cbl binds RET and MEN2A kinases. (**A**) HEK293T cells were transfected separately with pJ7Ω plasmids encoding each of the following four RET kinase isoforms: RET 9, RET 51, and their MEN2A (C634R) mutant isoforms. Each was transfected both with and without GST-Cbl-c. Whole cell lysates were collected at 48h post-transfection and GST pull-downs were performed on 300 µg of whole cell lysates and immunoblotted (IB) as indicated. Whole cell lysates were immunoblotted as indicated and molecular weight standards are indicated on the left of each panel β-actin serves as a loading control. (**B**) HEK293T cells were transfected with GST-Cbl-c both with and without RET9MEN2A. Replicate plates were treated with either 500 µM Sorafenib (+) or DMSO as vehicle control (-) for 90m prior to collection. Whole cell lysates were immunoblotted as indicated on the right. RET immunoprecipitations were performed on 300 µg of whole cell lysates using anti-RET antibody with Protein A/G Sepharose and immunoblotted for RET and phosphotyrosine (pY) as indicated. GST pull-downs were performed, as described above, and immunoblotted (IB) for RET, phosphotyrosine (pY), and GST-Cbl-c as indicated. Molecular weight standards are indicated to the left of each panel and Hsc70 serve as loading controls.

Enigma is known to bind RET directly through its LIM2 domain [19]. Cbl and Cbl-b, as negative regulators of RTKs, have been shown to bind to, ubiquitinate, and downregulate RET [26], [30]. The binding of Cbl and Cbl-b to RET is mediated by an indirect interaction where the Cbl protein binds to Grb2 or Shc which in turn bind to RET [26]. More recently, Cbl-c has been shown to interact with RET; however, the mechanism of binding and the physiological relevance of this interaction remain unclear [31]. There are several isoforms of RET which differ by alternative splicing of the C-terminus [32]. To confirm the interaction between Cbl-c and RET, we co-expressed GST-Cbl-c with the two most abundant isoforms of RET, RET9, RET51, and their corresponding constitutively active MEN2A (C634R) mutant isoforms. GST pull-downs confirmed the interaction between Cbl-c and each of the RET isoforms (Figure 2A, top panel, lanes 4, 6, 8, and 10). The interaction between Cbl-c and this RTK was further supported by the observation of tyrosine phosphorylation of Cbl-c in the presence of each RET isoform. This is indicated by the presence of a slower migrating Cbl-c band when RET is present (Figure 2A, third panel, compare lanes 4, 6, 8, and 10 to lane 2) and by direct immunoblotting of GST pull-downs for tyrosine phosphorylation (Figure 2A, second panel, compare lanes 4, 6, 8, and 10 to lane 2). To test whether this interaction was dependent upon RET activation, we employed Sorafenib, a multikinase inhibitor, which inhibits RET and is being used to currently treat RET-driven renal cancers [33], [34]. Cells treated with Sorafenib prior to lysis showed reduced RET phosphorylation indicating effective kinase inhibition (Figure 2B, top panel, lanes 5 and 6). GST pull-down of GST-Cbl-c demonstrated that despite effective RET inhibition, the interaction between Cbl-c and RET was maintained in the presence of Sorafenib (Figure 2B, third panel, lanes 3 and 6). These results suggest that this interaction is independent of RET phosphorylation. Immunoblotting also revealed a significant reduction of Cbl-c tyrosine phosphorylation in the presence of Sorafenib (Figure 2B, fifth panel, compare lanes 3 and 6) again consistent with RET dependent phosphorylation of Cbl-c.

### Cbl-c Enhances RET Ubiquitination

**Figure 3:**
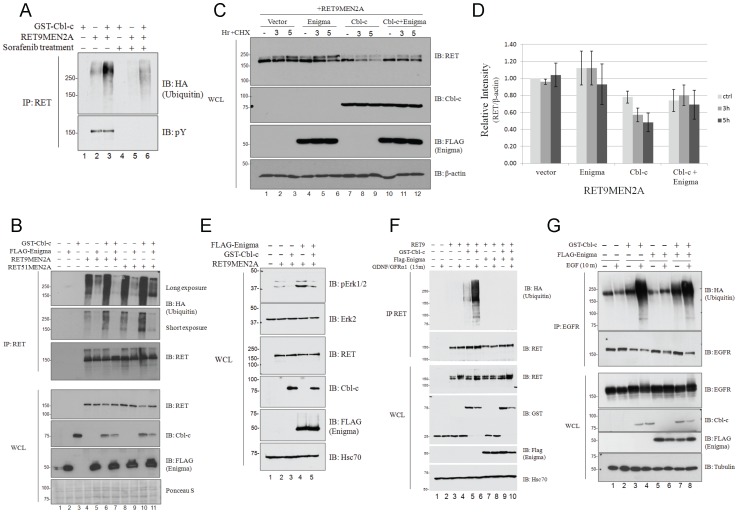
Enigma abrogates RETMEN2A ubiquitination. (**A**) RET immunoprecipitations were performed on 300 µg of each of the whole cell lysates described above and immunoblotted for HA-ubiquitin and phosphotyrosine (pY) as indicated on the right. (**B**) HEK293T cells were transfected with GST-Cbl-c with and without FLAG-Enigma along with either RET9MEN2A or RET51MEN2A. Cells were collected 48h post-transfection, and whole cell lysates were immunoblotted as indicated on the right. RET immunoprecipitations were performed on 300 µg of each of the whole cell lysates and immunoblotted for RET and HA-ubiquitin as indicated on the right. Molecular weight standards are indicated on the left and Ponceau S staining serves as a measure of protein loading. (**C**) HEK293T cells were transfected with RET9MEN2A with GST-Cbl-c and FLAG-Enigma both alone and in combination as indicated above the blots. All transfections were performed in triplicate. At 24h post-transfection, triplicate plates were pooled and replated. At 40h post-transfection, replicate plates were treated with 100ng/mL cycloheximide (CHX) for either 3 or 5h as indicated. Control plates received an equivalent volume of DMSO vehicle control for 5h prior to cell collection. A total of 20 µg of each of the whole cell lysates was immunoblotted as indicated. RET steady state levels were then assessed using β-actin as a loading control. (**D**) Densitometric analysis of RET steady state levels in the presence of GST-Cbl-c and Enigma, either alone or in combination. Levels were all compared to Vector transfected cells in the absence of cycloheximide. Error bars denote mean ± SE (n  = 3). (**E**) HEK293T cells were transfected with RET9MEN2A with GST-Cbl-c and FLAG-Enigma both alone and in combination as indicated above each blot. At 24h post-transfection, all cells were starved of FBS for 24h prior to cell harvesting. A total of 20 µgs of each of the whole cell lysates were immunoblotted as indicated to the right of each panel. Phospho-MAPK steady state levels were assessed using MAPK and Hsc70 as loading controls. Molecular weight standards are indicated on the left of each panel. (**F**) HEK293T cells were transfected with wild-type RET and the RET co-receptor, GFRα1 along with GST-Cbl-c and Enigma both alone and in combination. Vector controls included empty GST vector and all transfections were performed in duplicate and included HA-tagged ubiquitin. At 24h post-transfection, duplicate plates were pooled and re-plated to allow reattachment overnight. At 40h post-transfection, plates were rinsed and starved in media lacking FBS for 24h, then stimulated as described previously [43], using 30ng/mL GDNF and 100ng/mL exogenous GFRα1 (+) or water control (-) for 15m as indicated prior to collection and lysis. Whole cell lysates were immunoblotted as indicated. To assess RET ubiquitination, RET immunoprecipitations were performed on 300ugs of whole cell lysate and immunoblotted for HA-ubiquitin as indicated on the right. (**G**) HEK293T cells were transfected with EGFR along with GST-Cbl-c and Enigma both alone and in combination. All transfections were performed in duplicate. At 24h post-transfection, duplicate plates were pooled and replated to allow reattachment overnight. At 40h post-transfection, plates were rinsed and starved in media lacking FBS for 8h, then stimulated with 100ng/mL EGF (+) or water control (-) for 10m as indicated prior to collection and lysis. Whole cell lysates were immunoblotted as indicated. To assess EGFR ubiquitination, EGFR immunoprecipitations were performed on 300 µg of whole cell lysate and immunoblotted for EGFR and HA-ubiquitin as indicated on the right. Molecular weight standards are shown to the left of each panel and tubulin serves as loading control.

Like Cbl and Cbl-b, Cbl-c can ubiquitinate activated EGFR [35], [36]. While Cbl and Cbl-b are reported to negatively regulate RET [30], the ability of Cbl-c to ubiquitinate RET has not yet been clearly established. To test whether RET is a substrate of Cbl-c ubiquitin ligase function, we used the lysates described in Figure 2B in which we co-expressed GST-Cbl-c with the constitutively active mutant RET9MEN2A in HEK293T cells. To facilitate the detection of ubiquitinated RET, all transfections included an equal amount of HA-tagged ubiquitin [37]. We used the constitutively active RET9MEN2A isoform since this allows assessment of activated RET ubiquitination in the absence of both ligand and the RET co-receptor, GFRα [38]. Ubiquitination of RET9MEN2A in the presence and absence of GST-Cbl-c was assessed by RET immunoprecipitation and immunoblotting with and anti-HA HRP conjugated antibody. In the presence of GST-Cbl-c, RET9MEN2A showed enhanced ubiquitination indicating that Cbl-c can mediate ubiquitination of this constitutively active RET isoform (Figure 3A, top panel, compare lane 3 to lane 2). In addition to enhanced ubiquitination, protein steady state levels of both the receptor and Cbl-c were reduced when co-expressed suggesting enhanced and coordinated degradation of RET and Cbl-c (Figure 2B panels 7 and 8, lanes 1–3). Reduced steady state levels of Cbl-c in the presence of RET was consistent and most apparent in transfections with higher relative levels of RET to Cbl-c (not shown). While Sorafenib showed no effect on the ability of Cbl-c to bind RET (Figure 2B), ubiquitination was reduced in the presence of Sorafenib (Figure 3A, top panel, compare lane 6 to lane 3) indicating that, unlike Cbl-c recruitment, Cbl-c-mediated ubiquitination requires receptor activation. Interestingly, the downregulation of RET9MEN2A and GST-Cbl-c was attenuated in the presence of Sorafenib consistent with inhibition of downregulation of both proteins (Figure 2B, panels 7 and 8, compare lanes 4–6 to lanes 1–3).

### Enigma Abrogates RETMEN2A Ubiquitination and Degradation

Previously Enigma has been shown to bind RET at tyrosine 1062 in a phosphorylation independent manner [16], [20] and to be required for RET-mediated mitogenic signaling [16], [20]. Considering the interaction of Enigma with Cbl-c shown here and the ability of Cbl-c to ubiquitinate RET, we next tested whether the presence of Enigma had any effect on Cbl-c-mediated RET ubiquitination. Co-transfection of HEK293T cells with RET9MEN2A, RET51MEN2A, and GST-Cbl-c confirmed the enhanced ubiquitination of the activated RETMEN2A isoforms by Cbl-c (Figure 3B, top panel, compare lane 6 to lane 4 and lane 10 to lane 8). In the presence of Enigma, Cbl-c-mediated ubiquitination of both RETMEN2A isoforms was significantly reduced (Figure 3B, top panel compare lane 7 to lane 6 and lane 11 to lane 10). Enigma also reduced the ubiquitination of both RETMEN2A isoforms seen in the absence of GST-Cbl-c (Figure 3B, top panel, compare lane 5 to lane 4 and lane 9 to lane 8). The expression of Enigma also affected Cbl-c-mediated RET degradation (Figure 3C). Co-transfection studies, using the protein synthesis inhibitor, cycloheximide, showed reduced RET steady state levels in the presence of ectopic Cbl-c (Figure 3C, top panel, compare lanes 7–9 to lanes 1–3 and Figure 3D) indicating that Cbl-c ubiquitination promotes RET degradation. Densitometric analysis of RET band intensities from 3 independent experiments demonstrated that in the absence of Cbl-c (Figure 3C vector lanes 1–3 and Enigma lanes 4–6 and Figure 3D) there was no significant downregulation of the RET protein upon cycloheximide treatment. A consistent reduction in RET steady state levels in the presence of Cbl-c and further reduction upon cycloheximide treatment was observed (Figure 3C lanes 7–9 and Figure 3D). This is consistent with Cbl-c mediated ubiquitination and degradation of activated RET. In the presence of Enigma, RET steady state levels were reduced but the time dependent reduction upon cycloheximide treatment was not observed (Figure 3C lanes 10–12 and Figure 3D), consistent with the ability of Enigma to partially abrogate Cbl-c-mediated ubiquitination and subsequent downregulation of RET. In whole cell lysate immunoblots, RET appears as two distinct bands corresponding to the 175kD mature and 155kD immature forms [39], [40]. RET degradation, in the presence of ectopic Cbl-c, appears most dramatic on the larger, mature form (Figure 3C, top panel, lanes 7–9) indicating that Cbl-c mediates degradation of the mature, glycosylated form of the receptor. As with ubiquitination, the presence of Enigma blocks Cbl-c-mediated RET degradation (Figure 3C; compare lanes 10–12 to lanes 7–9). Enigma alone had little effect on RET steady state levels (Figure 3C, compare lanes 4–6 to lanes 1–3). RET9MEN2A activated downstream MAPK signaling in starved cells as evidenced by the increase in phospho-ERK 1/2 compared to starved cells without RET9MEN2A (Figure 3E; compare lanes 1 and 2). Phospho-ERK 1/2 was reduced when Cbl-c was expressed with RET9MEN2A, supporting the role of Cbl-c as a negative regulator of RET signaling (Figure 3E; compare lanes 2 and 3). Consistent with previously published work showing that Enigma is a positive regulator of oncogenic RET signaling [16], [20], ectopic expression of Enigma enhanced the activation of phospho-ERK by RET9MEN2A (Figure 3E; compare lanes 4 with lane 2). When Enigma and Cbl-c were co-expressed with RET9MEN2A, the phospho-ERK signaling was increased compared to cells transfected with Cbl-c and RET9MEN2A and in fact phospho-ERK was at the same level as seen in RET9MEN2A alone (Figure 3E; compare lane 5 to lanes 3 and 2 respectively). The enhanced MAPK signaling seen when Enigma is co-transfected with Cbl-c and RET9MEN2A is consistent with the inhibitory effects of Enigma on Cbl-c-mediated downregulation of RET.

Since these studies utilized the constitutively active, mutant form of RET, we tested whether Cbl-c and Enigma can similarly regulate ubiquitination of wild-type RET following ligand activation. To test this, we employed plasmids encoding wild-type RET9 and the RET co-receptor, GFRα1, which have been used successfully to assess GDNF induced activation previously in transfected cells [41]. Co-transfection of HEK293T cells with wild-type RET9 and GFRα1 along with GST-Cbl-c confirmed enhanced ubiquitination of wild-type RET9 following GDNF stimulation in ther presence of Cbl-c (Figure 3F, lanes 5–6). As illustrated with RETMEN2A, Cbl-c mediated ubiquitination of wild-type RET9 was abrogated in the presence of Enigma (Figure 3F, lanes 9–10).

To determine if the effect of Enigma on Cbl-c-mediated RET ubiquitination is a general phenomenon, we next tested whether Enigma similarly abrogated Cbl-c-mediated ubiquitination of activated EGFR. As previously demonstrated, Cbl-c enhanced EGFR ubiquitination upon EGF treatment (Figure 3G; top panel, compare lane 4 to lane 2). However, the presence of Enigma did not inhibit Cbl-c-mediated ubiquitination of activated EGFR (Figure 3G, top panel, compare lane 8 to lane 4).

**Figure 4:**
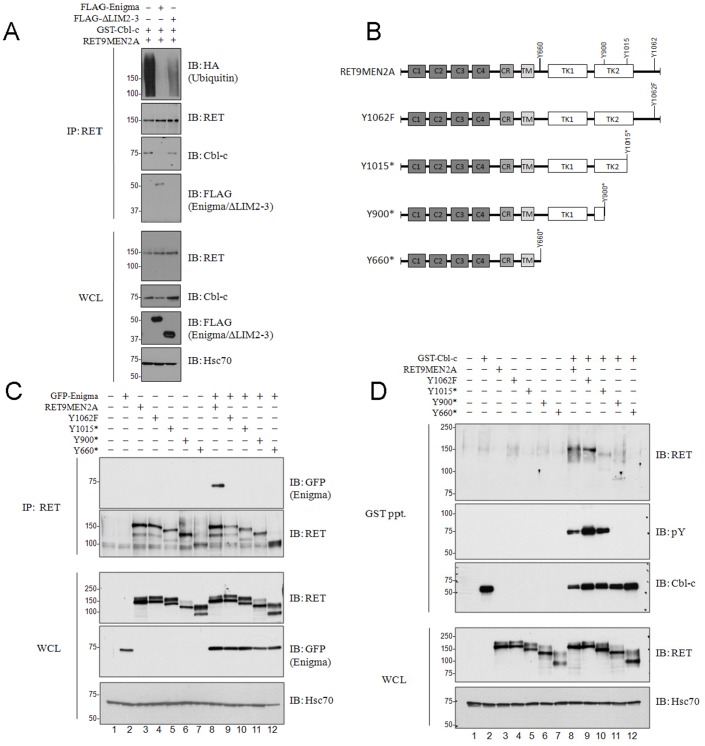
Enigma blocks Cbl-c-mediated RET9MEN2A ubiquitination. (**A**) HEK293T cells were transfected with RET9MEN2A along with GST-Cbl-c, FLAG-Enigma or FLAG-EnigmaΔLIM2-3 alone and in combination as indicated above the blots. All transfections included an equal amount of HA-ubiquitin. At 40h post-transfection, all cells were treated with 20nM MG-132 for 5h prior to cell collection. A total of 20 µg of each of the whole cell lysates were immunoblotted as indicated on the right. RET immunoprecipitations were performed on 300 µg of whole cell lysate and immunoblotted for RET and HA-ubiquitin. Co-immunoprecipitation of Cbl-c and Enigma were evaluated as indicated on the right. Molecular weight standards are indicated to the left of each panel and Hsc70 serves as a loading control. (**B**) RET truncation constructs used for mapping Cbl-c and Enigma interaction sites. Structural domains include the extracellular cadherin-like repeats (C1-4), a cysteine rich region (CR), a hydrophobic transmembrane region (TM), a split cytoplasmic tyrosine kinase domain (TK1 and TK2), and intracellular tyrosines subsequently mutated to an F or stop (*) to create each construct used in this study. (**C**) HEK293T cells were transfected with GFP-tagged Enigma both alone and in combination with RET9MEN2A or one of a series of RET9MEN2A mutant constructs as indicated above each blot. A total of 20 µg of each of the whole cell lysates were immunoblotted as indicated on the right. RET immunoprecipitations were performed on 300 µg of each of the whole cell lysates and immunoblotted for GFP-Enigma. Molecular weight standards are indicated on the left of each panel and Hsc70 serves as loading control. (**D**) HEK293T cells were transfected with each of the RET9MEN2A constructs both alone and in combination with GST-Cbl-c as indicated above each blot. All transfections were balanced with empty vector controls, and cells were collected 48 h post-transfection. A total of 20 µg of each of the whole cell lysates were immunoblotted as indicated on the right. GST pull-downs were performed on 300 µg of each of the whole cell lysates and immunoblotted for Cbl-c, phosphotyrosine (pY), and RET as indicated on the right. Molecular weight standards are indicated to the left of each panel and Hsc70 serves as a loading control.

To elucidate the mechanism by which Enigma inhibits Cbl-c-mediated ubiquitination of RET, we transfected HEK293T cells with RET9MEN2A, GST-Cbl-c, and either full-length FLAG-Enigma or a mutant lacking both the RET binding domain, LIM2 and LIM3 (ΔLIM2-3). As described above, the ΔLIM2-3 mutant maintains Cbl-c binding but lacks the RET binding site [19]. Immunoprecipitation of RETMEN9A confirmed that full length Enigma co-precipitated with RET9MEN2A while the ΔLIM2-3 mutant did not (Figure 4A, fourth panel, compare lane 2 and 3). As shown in Figure 3B, expression of Enigma with GST-Cbl-c inhibited Cbl-c mediated RET9MEN2A ubiquitination (Figure 4A, top panel, compare lanes 1 and 2). By contrast, co-transfection of ΔLIM2-3, which cannot bind to RET, resulted in significant recovery of ubiquitination of RET9MEN2A in the presence of GST-Cbl-c (Figure 4A, top panel, compare lane 3 to lanes 2). However, the Cbl-c-mediated ubiquitination of RET9MEN2A in the presence of ΔLIM2-3 was consistently less than that seen with Cbl-c alone suggesting that even when Enigma cannot bind to RET9MEN2A, it can still partially inhibit Cbl-c-mediated ubiquitination. Subsequent immunoblotting of RET9MEN2A immunoprecipitated proteins for GST-Cbl-c showed that co-immunoprecipitation of GST-Cbl-c with RET9MEN2A was inhibited when Enigma was present (Figure 4A, third panel, compare lane 2 to lane 1). ΔLIM2-3, which did not show any detectable co-precipitation with RET9MEN2A, also did not prevent the co-precipitation of GST-Cbl-c with RET.

The data in Figure 4A suggest that one mechanism by which Enigma prevents Cbl-c induced RET ubiquitination and downregulation is by blocking the binding of Cbl-c to RET. To determine whether Cbl-c and Enigma share the same binding regions on RET, we next mapped the binding of Enigma and Cbl-c to RET. To map the binding of Enigma to RET, HEK293T cells were co-transfected with wild-type and a series of mutant RET9MEN2A constructs (Figure 4B). GFP-tagged Enigma was employed to facilitate detection following RET immunoprecipitation since Enigma has a similar molecular weight of immunoglobulin proteins used for immunoprecipitation. Mutation of RET Y1062 to F or the deletion of any portion of the cytoplasmic tail that included Y1062 abrogated detectable Enigma co-immunoprecipitation with RET (Figure 4C; top panel, compare lane 8 to lanes 9–12), consistent with previous reports mapping RET Y1062 as the binding site for Enigma [16], [20]. To determine the binding site of Cbl-c on RET, HEK293T cells were co-transfected with GST-Cbl-c and the RET9MEN2A mutant constructs and GST pull-downs were performed. In contrast to the interaction between Enigma and RET9MEN2A, the association between Cbl-c and RET9MEN2A was maintained when Y1062 of RET9MEN2A was mutated to F (Figure 4D, top panel, compare lane 9 to lane 8). Deletion of the cytoplasmic tail by introducing a stop codon at Y1015 (Y1015*) of RET9MEN2A resulted in decreased binding of Cbl-c but not complete loss of binding (Figure 4D, top panel, compare lane 10 to lane 8). Similarly, introducing a stop codon at Y900 (Y900*) of RET9MEN2A resulted in decreased binding of Cbl-c compared to the full length RET9MEN2A (Figure 4D, top panel, compare lane 11 to lane 8). However, there was still detectable binding similar to the Y1015* truncation (Figure 4D, top panel, compare lane 11 to lane 10). Truncation of RET9MEN2A near the membrane (Y600*) abrogated the interaction between Cbl-c and RET9MEN2A (Figure 4D, top panel, compare lane 12 to lane 8). Thus, while Enigma binds to the RET9MEN2A by an interaction mediated by Y1062, Cbl-c does not. Instead, Cbl-c interacts with RET9MEN2A through at least two regions, one between Y1015 and the end of the protein and another between the membrane and Y1015.

### Cbl-c and Enigma Expression have Opposing Correlations with Survival Outcome

**Figure 5:**
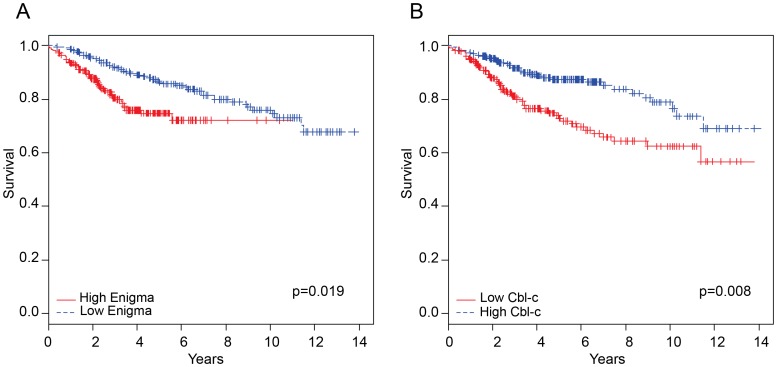
Comparison of survival outcome between Enigma and Cbl-c expression in high RET expressing breast cancers. Kaplan-Meier survival analysis over time with high vs. low Enigma expression (**A**) and high vs. low Cbl-c expression (**B**). The P-value was determined by log-rank test between risk groups.

While activating RET mutations are associated with thyroid carcinomas and multiple endocrine neoplasias, there are no publicly available datasets that would allow us to correlate clinical outcome with the expression of Enigma and Cbl-c. However, several recent studies have shown that RET is expressed in a subset of breast cancers [42]–[44] and that high RET expression is associated with poor prognosis [45]. To assess the effects of Enigma and Cbl-c expression on outcomes in patients whose tumors express RET, we combined two publicly available breast cancer datasets for which RET, Cbl-c and Enigma expression and outcome data were available [27], [28]. Both studies included patients with early stage breast cancer treated with chemotherapy. The merged cohort consisted of 835 samples. Based upon a median cut-off for expression of RET across these samples, a subset of 459 patients were extracted that had higher than median levels of RET expression. Among those with high RET expression, patients expressing high levels of Enigma showed reduced survival (Figure 5A). By contrast, patients expressing high levels of Cbl-c showed prolonged survival (Figure 5B). The survival of patients with low levels of RET expression showed no statistically significant correlation with the expression of Cbl-c or Enigma.

## Discussion

RET is an RTK which binds members of the glial cell line-derived neurotrophic factor (GDNF) family of ligands [21]. RET gain of function mutations and translocations are associated with the development of a variety of human cancers, including medullary thyroid carcinoma, multiple endocrine neoplasias (MEN) type 2A and 2B, pheochromocytoma, and parathyroid hyperplasia [23], [24]. Also previous reports have demonstrated the LIM protein Enigma as a required component for mitogenic signaling of RET/PTC [16], a rearranged oncogenic isoform of RET found in papillary thyroid carcinomas. Cbl proteins are a family of ubiquitin ligases that negatively regulate RTKs, including RET [1], [2]. Using a yeast two-hybrid screen, we identified Enigma as a new Cbl-c interacting protein (Figure 1) which abrogates Cbl-c-mediated ubiquitination and downregulation of RET9MEN2A, a RET isoform associated with multiple endocrine neoplasia (MEN) type 2A.

Enigma is a member of the LIM family of proteins, consisting of an N-terminal PDZ domain and three C-terminal LIM domains, which mediate protein-protein interactions. This LIM protein is considered a scaffold protein through which coordinated assembly of proteins can occur and has been implicated as an adapter that localizes LIM-binding proteins to actin filaments through its PDZ domain [17]. Enigma has three LIM domains. LIM1 is the most structurally diverse and has no known interacting partners [29] while LIM2 and LIM3 bind RET and InsR respectively [19], [20]. The interaction between Cbl-c and Enigma appears to be LIM-mediated as deletion of the C-terminus, which contains LIM1-3, abrogates this interaction while the region including LIM1 is sufficient for binding Cbl-c (Figure 2). This interaction is specific to Cbl-c as Enigma did not co-precipitate with either Cbl or Cbl-b (Figure 2). The yeast two-hybrid screen used the C-terminal portion of Cbl-c as bait. The three human Cbl proteins are most divergent through their C-termini and these differences likely account for the specificity of Enigma binding to Cbl-c.

Like Cbl and Cbl-b, Cbl-c is an E3 which has been shown to ubiquitinate and downregulate activated RTKs (*e.g.*, EGFR) and non-receptor TKs (*e.g.* Src) [11], [35], [36]. In addition to its adaptor function, Enigma has been reported to bind and modulate the E3 function of MDM2 [46]. MDM2 can auto-ubiquitinate leading to proteasomal degradation of MDM2 [47], [48]. Also, MDM2 binds to and ubiquitinates p53 resulting in degradation of p53 [47]. Recently it has been shown that binding of Enigma enhances ubiquitination of p53 while reducing auto-ubiquitination thereby enhancing p53 degradation [46]. Thus, we tested whether Enigma could modulate the E3 activity of Cbl-c. Co-expression of Enigma appeared to have little to no detectable effect on the ability of Cbl-c to ubiquitinate activated EGFR (Figure 2). This suggests that binding of Enigma to Cbl-c does not directly affect its E3 activity.

Cbl proteins have been shown to bind to and downregulate RET, an RTK which is activated by glial cell derived neurotrophic factor family ligands. RET is involved in numerous cellular processes including proliferation, neuronal navigation, migration, and differentiation. Gain of function mutations of RET are responsible for most cases of MEN2. Here, we show that Cbl-c can bind the wild-type RET9, RET51, and the mutant MEN2A isoforms (Figure 3). Precipitated Cbl-c is tyrosine phosphorylated in the presence of RET suggesting that Cbl-c may be a substrate of RET kinase function. While treatment with Sorafenib, a known RET inhibitor, does block Cbl-c phosphorylation, it remains unclear whether RET is directly phosphorylating Cbl-c or if other kinases downstream of RET are responsible for Cbl-c phosphorylation. Cbl and Cbl-b binding to activated RET is indirect and mediated by Shc and Grb2, which bind to Y1062 and Y1096 on RET respectively. The shorter RET9 lacks Y1096 and this may be at least in part why Cbl and Cbl-b show preferential association with the longer RET51 isoform [26]. Cbl-c binds both isoforms and their MEN2A mutants in the absence of ligand activation or co-receptor expression suggesting that Cbl-c recruitment may be constitutive and independent of the RET isoform. Treatment with Sorafenib did not block the association of Cbl-c with the RET isoforms, further supporting a constitutive interaction. However, Sorafenib treatment did block phosphorylation of Cbl-c and Cbl-c-mediated ubiquitination of RET indicating that, while association is constitutive, ubiquitination is dependent upon receptor activation. In addition to enhanced ubiquitination, protein steady state levels of both the receptor and Cbl-c were consistently reduced when co-expressed suggesting enhanced and coordinated degradation of RET and Cbl-c (Figure 2B). Coordinated degradation of Cbl proteins with their RTK substrates has been previously reported by our lab and others and is consistent with an interaction between these two proteins [49], [50].

Enigma has been shown to bind to RET and is required for mitogenic signaling of an oncogenic form of RET [19], [20]. Interestingly, Enigma blocks Cbl-c-mediated RETMEN2A ubiquitination, downregulation (Figure 3B-D and 4A) and promotes RET signaling through MAPK (Figure 3E). The ability of Enigma to abrogate Cbl-c-mediated downregulation of RET likely accounts for the increase in MAPK phosphorylation observed in the presence of Enigma (Figure 3E). Our data suggest that Enigma inhibits Cbl-c-mediated RETMEN2A ubiquitination at least in part by preventing Cbl-c binding to RET (Figure 4C). We show here that the opposing actions of Cbl-c and Enigma demonstrated on RETMEN2A ubiquitination is conserved in ligand activated wild-type RET9 (Figure 3F), and that this effect is specific to RET as Enigma does not similarly abrogate Cbl-c-mediated ubiquitination of activated EGFR (Figure 3G). Through mapping of the RET binding sites for each of these proteins, we show that Enigma and Cbl-c bind at different sites within the intracellular portion of RET (Figure 4). As previously reported, a point mutation or deletion of residue Y1062 of RET disrupts all detectable interaction between RET and Enigma [16], [20]. Based on our mapping with a series of truncation mutants, RET-Cbl-c binding is maintained in the Y1062 mutant and is mediated through several sites within the intracellular domain of RET (Figure 4). This suggests that the inhibition of Cbl-c binding to RET by Enigma is through steric hindrance and not *via* a direct competition for the binding site. However, the data also suggest that some of the effect of Enigma on Cbl-c-mediated RET ubiquitination is *via* sequestration of Cbl-c by Enigma. This is supported by the only partial rescue of Cbl-c-mediated RET ubiquitination when Enigma ΔLIM2-3 (which lacks the RET binding site but maintains Cbl-c binding) is expressed along with Cbl-c. If Enigma was acting solely through competitive binding of RET, then the deletion of LIM2 should completely restore Cbl-c-mediated RET ubiquitination.

RET expression is associated with poor outcome in a subset of breast cancers [45]. In a merged dataset of breast cancer outcome following early treatment [27], [28], high Cbl-c expression correlated with increased survival, while high Enigma expression correlated with reduced survival (Figure 5). The contrasting effects of expression on clinical outcome in high RET breast cancers support the role of Cbl-c as a negative regulator of RET signaling and of Enigma opposing this action and potentiating RET signaling.

Overall, our data demonstrate the interaction between a positive and negative regulator of an oncogenic form of RET. We show that Cbl-c negatively regulates RET by ubiquitinating and downregulating the activated RTK while Enigma positively regulates activated RET by preventing Cbl-c binding to RET and thus preventing RET ubiquitination and degradation while promoting RET mitogenic signaling. This is supported by clinical data among high RET expressing breast cancers. In light of recent data demonstrating the role of Enigma in modulating degradation of p53, this data illustrates another mechanism highlighting the oncogenic nature of Enigma. Understanding this interaction, and its role in RTK regulation, has potential implications in further understanding RET-driven tumorigenesis.

## References

[1] Nau MM, Lipkowitz S (2003) Comparative genomic organization of the cbl genes. Gene 308: 103–113. 12711395 10.1016/s0378-1119(03)00471-2

[2] Nau MM, Lipkowitz S (2008) Welcome to the Family: Cbl-Famil Gene Organization, Overview of Structure and Functions of Cbl-Related Proteins in Various Taxonomical Groups. In: Tsygankov AY, editor. Cbl Proteins. 1 ed. New York: Nova Science Publishers, Inc. 3–25.

[3] Schmidt MH, Dikic I (2005) The Cbl interactome and its functions. Nat Rev Mol Cell Biol 6: 907–918. 16227975 10.1038/nrm1762

[4] Davies GC, Ettenberg SA, Coats AO, Mussante M, Ravichandran S, et al (2004) Cbl-b interacts with ubiquitinated proteins; differential functions of the UBA domains of c-Cbl and Cbl-b. Oncogene 23: 7104–7115. 15273720 10.1038/sj.onc.1207952

[5] Peschard P, Kozlov G, Lin T, Mirza IA, Berghuis AM, et al (2007) Structural basis for ubiquitin-mediated dimerization and activation of the ubiquitin protein ligase Cbl-b. Mol Cell 27: 474–485. 17679095 10.1016/j.molcel.2007.06.023

[6] Griffiths EK, Sanchez O, Mill P, Krawczyk C, Hojilla CV, et al (2003) Cbl-3-deficient mice exhibit normal epithelial development. Mol Cell Biol 23: 7708–7718. 14560016 10.1128/MCB.23.21.7708-7718.2003 PMC207562

[7] Keane MM, Ettenberg SA, Nau MM, Banerjee P, Cuello M, et al (1999) cbl-3: a new mammalian cbl family protein. Oncogene 18: 3365–3375. 10362357 10.1038/sj.onc.1202753

[8] Keane MM, Rivero-Lezcano OM, Mitchell JA, Robbins KC, Lipkowitz S (1995) Cloning and characterization of cbl-b: a SH3 binding protein with homology to the c-cbl proto-oncogene. Oncogene 10: 2367–2377. 7784085

[9] Langdon WY, Blake TJ (1990) The human CBL oncogene. Curr Top Microbiol Immunol 166: 159–164. 2073794 10.1007/978-3-642-75889-8_20

[10] Levkowitz G, Waterman H, Ettenberg SA, Katz M, Tsygankov AY, et al (1999) Ubiquitin ligase activity and tyrosine phosphorylation underlie suppression of growth factor signaling by c-Cbl/Sli-1. Mol Cell 4: 1029–1040. 10635327 10.1016/s1097-2765(00)80231-2

[11] Kim M, Tezuka T, Tanaka K, Yamamoto T (2004) Cbl-c suppresses v-Src-induced transformation through ubiquitin-dependent protein degradation. Oncogene 23: 1645–1655. 14661060 10.1038/sj.onc.1207298

[12] Bachmaier K, Krawczyk C, Kozieradzki I, Kong YY, Sasaki T, et al (2000) Negative regulation of lymphocyte activation and autoimmunity by the molecular adaptor Cbl-b. Nature 403: 211–216. 10646608 10.1038/35003228

[13] Chiang YJ, Kole HK, Brown K, Naramura M, Fukuhara S, et al (2000) Cbl-b regulates the CD28 dependence of T-cell activation. Nature 403: 216–220. 10646609 10.1038/35003235

[14] Murphy MA, Schnall RG, Venter DJ, Barnett L, Bertoncello I, et al (1998) Tissue hyperplasia and enhanced T-cell signalling via ZAP-70 in c-Cbl-deficient mice. Mol Cell Biol 18: 4872–4882. 9671496 10.1128/mcb.18.8.4872 PMC109072

[15] Naramura M, Kole HK, Hu RJ, Gu H (1998) Altered thymic positive selection and intracellular signals in Cbl-deficient mice. Proc Natl Acad Sci U S A 95: 15547–15552. 9861006 10.1073/pnas.95.26.15547 PMC28080

[16] Durick K, Gill GN, Taylor SS (1998) Shc and Enigma are both required for mitogenic signaling by Ret/ptc2. Mol Cell Biol 18: 2298–2308. 9528800 10.1128/mcb.18.4.2298 PMC121481

[17] Guy PM, Kenny DA, Gill GN (1999) The PDZ domain of the LIM protein enigma binds to beta-tropomyosin. Mol Biol Cell 10: 1973–1984. 10359609 10.1091/mbc.10.6.1973 PMC25398

[18] Bach I (2000) The LIM domain: regulation by association. Mech Dev 91: 5–17. 10704826 10.1016/s0925-4773(99)00314-7

[19] Wu RY, Gill GN (1994) LIM domain recognition of a tyrosine-containing tight turn. J Biol Chem 269: 25085–25090. 7929196

[20] Durick K, Wu RY, Gill GN, Taylor SS (1996) Mitogenic signaling by Ret/ptc2 requires association with enigma via a LIM domain. J Biol Chem 271: 12691–12694. 8662982 10.1074/jbc.271.22.12691

[21] Takahashi M, Cooper GM (1987) ret transforming gene encodes a fusion protein homologous to tyrosine kinases. Mol Cell Biol 7: 1378–1385. 3037315 10.1128/mcb.7.4.1378-1385.1987 PMC365224

[22] Parisi MA, Kapur RP (2000) Genetics of Hirschsprung disease. Curr Opin Pediatr 12: 610–617. 11106284 10.1097/00008480-200012000-00017

[23] Hansford JR, Mulligan LM (2000) Multiple endocrine neoplasia type 2 and RET: from neoplasia to neurogenesis. J Med Genet 37: 817–827. 11073534 10.1136/jmg.37.11.817 PMC1734482

[24] Gujral TS, Mulligan LM (2006) Molecular implications of RET mutations for pheochromocytoma risk in multiple endocrine neoplasia 2. Ann N Y Acad Sci 1073: 234–240. 17102091 10.1196/annals.1353.025

[25] Ettenberg SA, Keane MM, Nau MM, Frankel M, Wang LM, et al (1999) cbl-b inhibits epidermal growth factor receptor signaling. Oncogene 18: 1855–1866. 10086340 10.1038/sj.onc.1202499

[26] Scott RP, Eketjall S, Aineskog H, Ibanez CF (2005) Distinct turnover of alternatively spliced isoforms of the RET kinase receptor mediated by differential recruitment of the Cbl ubiquitin ligase. J Biol Chem 280: 13442–13449. 15677445 10.1074/jbc.M500507200

[27] Hatzis C, Pusztai L, Valero V, Booser DJ, Esserman L, et al (2011) A genomic predictor of response and survival following taxane-anthracycline chemotherapy for invasive breast cancer. JAMA 305: 1873–1881. 21558518 10.1001/jama.2011.593 PMC5638042

[28] Kao KJ, Chang KM, Hsu HC, Huang AT (2011) Correlation of microarray-based breast cancer molecular subtypes and clinical outcomes: implications for treatment optimization. BMC Cancer 11: 143. 21501481 10.1186/1471-2407-11-143 PMC3094326

[29] Wu R, Durick K, Songyang Z, Cantley LC, Taylor SS, et al (1996) Specificity of LIM domain interactions with receptor tyrosine kinases. J Biol Chem 271: 15934–15941. 8663233 10.1074/jbc.271.27.15934

[30] Pierchala BA, Milbrandt J, Johnson EM Jr (2006) Glial cell line-derived neurotrophic factor-dependent recruitment of Ret into lipid rafts enhances signaling by partitioning Ret from proteasome-dependent degradation. J Neurosci 26: 2777–2787. 16525057 10.1523/JNEUROSCI.3420-05.2006 PMC6675173

[31] Tsui CC, Pierchala BA (2008) CD2AP and Cbl-3/Cbl-c constitute a critical checkpoint in the regulation of ret signal transduction. J Neurosci 28: 8789–8800. 18753381 10.1523/JNEUROSCI.2738-08.2008 PMC3844776

[32] Tahira T, Ishizaka Y, Itoh F, Sugimura T, Nagao M (1990) Characterization of ret proto-oncogene mRNAs encoding two isoforms of the protein product in a human neuroblastoma cell line. Oncogene 5: 97–102. 2181380

[33] Plaza-Menacho I, Mologni L, Sala E, Gambacorti-Passerini C, Magee AI, et al (2007) Sorafenib functions to potently suppress RET tyrosine kinase activity by direct enzymatic inhibition and promoting RET lysosomal degradation independent of proteasomal targeting. J Biol Chem 282: 29230–29240. 17664273 10.1074/jbc.M703461200

[34] Santarpia L, Bottai G (2013) Inhibition of RET Activated Pathways: Novel Strategies for Therapeutic Intervention in Human Cancers. Curr Pharm Des 19: 864–882. 22973956

[35] Davies GC, Ryan PE, Rahman L, Zajac-Kaye M, Lipkowitz S (2006) EGFRvIII undergoes activation-dependent downregulation mediated by the Cbl proteins. Oncogene 25: 6497–6509. 16702950 10.1038/sj.onc.1209662 PMC2274962

[36] Ryan PE, Kales SC, Yadavalli R, Nau MM, Zhang H, et al (2012) Cbl-c ubiquitin ligase activity is increased via the interaction of its RING finger domain with a LIM domain of the paxillin homolog, Hic 5. PLoS One 7: e49428. 23145173 10.1371/journal.pone.0049428 PMC3492284

[37] Treier M, Staszewski LM, Bohmann D (1994) Ubiquitin-dependent c-Jun degradation in vivo is mediated by the delta domain. Cell 78: 787–798. 8087846 10.1016/s0092-8674(94)90502-9

[38] Borrello MG, Smith DP, Pasini B, Bongarzone I, Greco A, et al (1995) RET activation by germline MEN2A and MEN2B mutations. Oncogene 11: 2419–2427. 8570194

[39] Richardson DS, Rodrigues DM, Hyndman BD, Crupi MJ, Nicolescu AC, et al (2012) Alternative splicing results in RET isoforms with distinct trafficking properties. Mol Biol Cell 23: 3838–3850. 22875993 10.1091/mbc.E12-02-0114 PMC3459860

[40] Takahashi M, Asai N, Iwashita T, Isomura T, Miyazaki K, et al (1993) Characterization of the ret proto-oncogene products expressed in mouse L cells. Oncogene 8: 2925–2929. 8414495

[41] Trupp M, Raynoschek C, Belluardo N, Ibanez CF (1998) Multiple GPI-anchored receptors control GDNF-dependent and independent activation of the c-Ret receptor tyrosine kinase. Mol Cell Neurosci 11: 47–63. 9608533 10.1006/mcne.1998.0667

[42] Tozlu S, Girault I, Vacher S, Vendrell J, Andrieu C, et al (2006) Identification of novel genes that co-cluster with estrogen receptor alpha in breast tumor biopsy specimens, using a large-scale real-time reverse transcription-PCR approach. Endocr Relat Cancer 13: 1109–1120. 17158757 10.1677/erc.1.01120

[43] Boulay A, Breuleux M, Stephan C, Fux C, Brisken C, et al (2008) The Ret receptor tyrosine kinase pathway functionally interacts with the ERalpha pathway in breast cancer. Cancer Res 68: 3743–3751. 18483257 10.1158/0008-5472.CAN-07-5100

[44] Esseghir S, Todd SK, Hunt T, Poulsom R, Plaza-Menacho I, et al (2007) A role for glial cell derived neurotrophic factor induced expression by inflammatory cytokines and RET/GFR alpha 1 receptor up-regulation in breast cancer. Cancer Res 67: 11732–11741. 18089803 10.1158/0008-5472.CAN-07-2343

[45] Wang C, Mayer JA, Mazumdar A, Brown PH (2012) The rearranged during transfection/papillary thyroid carcinoma tyrosine kinase is an estrogen-dependent gene required for the growth of estrogen receptor positive breast cancer cells. Breast Cancer Res Treat 133: 487–500. 21947652 10.1007/s10549-011-1775-9 PMC3424514

[46] Jung CR, Lim JH, Choi Y, Kim DG, Kang KJ, et al (2010) Enigma negatively regulates p53 through MDM2 and promotes tumor cell survival in mice. J Clin Invest 120: 4493–4506. 21060154 10.1172/JCI42674 PMC2993588

[47] Fang S, Jensen JP, Ludwig RL, Vousden KH, Weissman AM (2000) Mdm2 is a RING finger-dependent ubiquitin protein ligase for itself and p53. J Biol Chem 275: 8945–8951. 10722742 10.1074/jbc.275.12.8945

[48] Lipkowitz S, Weissman AM (2011) RINGs of good and evil: RING finger ubiquitin ligases at the crossroads of tumour suppression and oncogenesis. Nat Rev Cancer 11: 629–643. 21863050 10.1038/nrc3120 PMC3542975

[49] Ettenberg SA, Magnifico A, Cuello M, Nau MM, Rubinstein YR, et al (2001) Cbl-b-dependent coordinated degradation of the epidermal growth factor receptor signaling complex. J Biol Chem 276: 27677–27684. 11375397 10.1074/jbc.M102641200

[50] Zeng S, Xu Z, Lipkowitz S, Longley JB (2005) Regulation of stem cell factor receptor signaling by Cbl family proteins (Cbl-b/c-Cbl). Blood 105: 226–232. 15315962 10.1182/blood-2004-05-1768

